# Contraceptive use, knowledge, attitude, perceptions and sexual behavior among female University students in Uganda: a cross-sectional survey

**DOI:** 10.1186/s12905-016-0286-6

**Published:** 2016-01-27

**Authors:** Henry Nsubuga, Juliet N. Sekandi, Hassard Sempeera, Fredrick E. Makumbi

**Affiliations:** 1 Makerere University, Counseling and Guidance Centre, Kampala, Uganda; 2 Makerere University, College of Health Sciences, School of Public Health, Kampala, Uganda; 3 Department of Epidemiology and Biostatistics, College of Public Health, University of Georgia, Athens, Georgia USA

**Keywords:** Contraceptives, Female undergraduates, Knowledge, Attitudes, Perceptions

## Abstract

**Background:**

In Uganda, the risk of unintended pregnancies and unsafe abortions remains high due to relatively low contraceptive use. There is paucity of data on knowledge, attitudes, perceptions and practices towards modern contraceptives and, sexual and reproductive health especially among the young female university students.

**Methods:**

A survey was conducted at Makerere University main campus in Kampala, Uganda during April 2014. A team of well-trained and experienced research assistants interviewed female undergraduate students who provided data on socio-demographic characteristics, knowledge, perceptions and attitudes and use of contraceptives, as well as other sexual and reproductive health practices. Users of any contraceptive method in the past 12 months were coded as ‘1’ and none users as ‘0’. The prevalence of contraceptive use was determined as the number of users divided by all female participants. Prevalence ratios (PRs) with their corresponding 95 % confidence intervals were used as measures of association between contraceptive use and associated factors. The PRs were obtained via a modified Poisson regression model using a generalized linear model with Poisson as family and a log link without an offset but including robust standard errors. All analyses were conducted with Stata version 13.

**Results:**

A total of 1,008 females responded to the survey; median (IQR) age was 21(20, 21) years, 38.6 % in year 2 of study, and nearly three quarters (72.3 %) were of Christian faith. Knowledge of any contraceptives was almost universal (99.6 %) but only 22.1 % knew about female condoms. Perceived acceptability of contraceptive use at the university (93 %) or being beneficial to male partners too (97.8 %) were high. Nearly 70 % had ever engaged in sexual intercourse and 62.1 % reported sexual intercourse in the past 12 months. Overall, 46.6 % reported current contraceptive use, with male condoms (34.5 %) being the commonest methods. Factors associated with higher contraceptive use were being in year 2, consensual union or perception that contraceptives are for females only. However, being evangelical/SDA or perception that contraceptive use is wrong was associated with lower contraceptive use. Overall, 9 % reported ever being pregnant, 2 % were pregnant at the time of the survey and a third (33.8 %) knew of a pregnant friend. About 40 % of ever pregnant respondents reported ever trying to terminate the pregnancy.

**Conclusions:**

Knowledge, perceived acceptability and benefits of contraceptive use were nearly universal, but contraceptive use was suboptimal in this setting. Ever trying to terminate a pregnancy was common and a clear indicator of unintended pregnancies.

## Background

The proportion of young women reporting unintended pregnancy and unmet need for contraception remains high in developing countries [1]. Unintended pregnancies are associated with increased risk of unsafe abortions, maternal morbidity and mortality [2]. In order to avert the unintended pregnancies and consequent adverse outcomes, contraceptive use has been prioritized as a key intervention [3]. Improving the universal access to sexual and reproductive health services including contraceptives was a key target of the Millennium Development Goals (MDG) [4, 5].

In developing countries, one in three women give birth before the age of 20 and pregnancy-related death during child birth is two times higher compared to women older than 20 years [5]. A quarter of the estimated 20 million unsafe abortions and 70,000 related deaths each year occur among women aged 15–19 years [5]. In sub-Saharan Africa alone, it is estimated that 14 million unintended pregnancies occur every year, with almost half occurring among women aged 15–24 years [6]. It is evident that use of effective contraceptive methods would potentially prevent 90 % of abortions, 20 % pregnancy-related morbidity and a third (32 %) of maternal deaths worldwide [4].

In Uganda, an estimated 1.2 million unintended pregnancies occurred in 2008, representing more than half of the country’s 2.2 million pregnancies [2]. The risk of pregnancy increases with a widening gap between sexual debut and age of first marriage [7, 8]. In Uganda nearly two thirds (64 %) of women aged 25–49 years reported early sexual debut before the age of 18 years [9]. At the time of enrolling into Universities, women are at an age of about two years above the median age of sexual debut in Uganda suggesting that they are usually sexually active.

Overall, the use of contraceptives is not openly discussed among young unmarried women due to strong cultural and religious beliefs, which exposes the young women to the increased risk of unwanted/unintended pregnancies. In many African traditional culture settings, pregnancy before marriage is often viewed as an abomination. As such, many unmarried females who get unintended pregnancies seek abortions services for fear of societal judgment. Abortion in Uganda being illegal increases the risk of maternal deaths because it is usually unsafe and at times conducted by traditional herbalists.

According to the two major surveys conducted among university students in Uganda, findings indicated that students did not have access to sexual and reproductive health services and HIV/AIDS-related programmes despite their engagement in high-risk sexual behaviours [10, 11]. Findings also showed that a quarter (25 %) of the university students had unmet need for contraceptives yet their level of awareness about contraceptives was high [1, 12]. Knowledge, attitudes and perceptions (KAP) about sexual and reproductive health may influence contraceptive use suggesting that interventions based on KAP may lead to reduced rates of unintended pregnancies [13].

This study was motivated by the continued anecdotal reports showing a common occurrence of unintended pregnancies and sexually transmitted infections among students seeking services at the University’s Counseling and Guidance Centre. However, there was paucity of data on the knowledge, attitudes, and perceptions, access to and use of contraceptives and sexual and reproductive health services/information among Makerere University female students. Data generated from this study will inform and enhance the design of student-centered programs to improve contraceptive knowledge and use, and thus potentially avert the unintended pregnancies and the consequent adverse outcomes in teaching institutions such as Makerere University.

## Methods

### Design, study setting and population

Across-sectional survey was conducted among female undergraduate students at Makerere University main campus. Makerere University is the oldest and largest public institution of higher education in Uganda. The University offers both undergraduate and graduate programs to approximately 40,000 students, half of who are females. The main university campus is located about 2.5 km from the capital city, Kampala.

Data were collected from April 1^st^ to April 30^th^ 2014 among 1,008 students selected on the basis of halls of residence using quantitative data collection tools. All registered undergraduate students aged 18–30 years were eligible to participate in the study. Some female students resided at the three halls of residence at the main campus while majority resided off-campus at private hostels and a few were commuting from their parents’/guardians’ homes.

The study outcomes included knowledge, perception/attitudes towards contraception, and reported contraceptive use in the 12 months following the survey. The independent variables were age, year of study, religion/faith, marital status and source of health information about family planning services and commodities.

Knowledge was defined as the state of awareness of contraceptive methods, any specific types and the source of contraceptives. Attitude or perception was defined as respondent’s opinion or view, whether positive or negative towards a practice or behavior such as contraceptive use. Knowledge was operationalized based on the following question “*Have you ever heard about contraceptives?*” Affirmative responses (yes) led to subsequent questions on the reasons for use of contraceptives, methods and the sources known to her. Then the respondent was considered knowledgeable if they correctly provided responses based on the listed options on the tool. The perceptions/attitudes of respondents about contraceptives were assessed using a 5 point-likert scale consisting of a range of responses from strongly agree (1), agree (2), neutral (3), disagree (4) and strongly disagree (5). Statements such as “*It is not easy for me to discuss with my partner about sexual matters*”, “*Family planning is for females only and not males*”, “*Family planning is acceptable in our community*” were used to elicit respondent’s views/options so as to determine their perceptions or attitudes towards family planning.

### Sample size estimation

A total of 973 female students were estimated as the appropriate sample size for this survey using the modified Kish-Leslie formula (Leslie Kish, 1965) for cross sectional studies. The sample size determination assumed 21.5 % of female students using modern contraceptives, a 5 % margin of error for the estimated p, 95 % confidence interval around the estimates, a design-effect of 3 to account for multistage sampling conducted through use of residential halls as clusters, and a survey response rate of 80 %. However, a total of 1,008 students were interviewed creating an excess of 35 respondents because interviews were conducted simultaneously at the multiple sites within the University.

### Participant recruitment

The initial participant recruitment plan included a sampling frame of about 22,000 female registered students as of April 2014, which was obtained from the university academic registrar’s office. The list included each student’s assigned hall of residence, year of study, faculty where registered for the studies, and telephone contacts. A sample of 973 students was randomly selected from this sampling frame. However, only eighty-five students could be reached via the registered telephone contacts. Reasons for none accessibility was primarily due to incorrectly registered telephone numbers or use of the next of kin telephone number who was not aware of the student’s whereabouts at the time of the survey. Although three callbacks were conducted, this approach did not result into any improvement in response rates. The research team thereafter made a protocol amendment to recruit participants using convenience sampling. Interviewers were therefore assigned or stationed at the various academic units within the university during daytime (8 hours) to request students to participant in the study.

### Data collection and quality assurance

The data collection tools were pre-tested and the study protocol piloted using twenty (20) questionnaires at a university within Kampala with similar characteristics as Makerere University. Research assistants/Interviewers were trained in data collection, research ethics, interviewer skills, seeking and administering informed consent. All the interviewers were graduates because the undergraduate participants were perceived to respond to persons who were senior in academic training. The interviews were conducted in English which both the interviewers and participants were knowledgeable. All completed questionnaires were reviewed by the field supervisors and checked for completeness by data editor at Makerere University School of Public Health data management centre. Interviewers were only allowed to interview another respondent after the field supervisor had completed reviewing the completed questionnaire.

### Ethics statement

This study was reviewed and received ethical approval from the Higher Degrees Research and Ethics Committee at Makerere University School of Public Health and the Uganda National Council for Science and Technology. Permission to conduct the study was granted by the Makerere University administration. Written informed consent was obtained from all study participants.

### Statistical analysis

Exploratory data analysis was conducted on all key variables. Descriptive statistics were generated with proportions (or percentages) for categorical data, and mean (standard deviation) for normally distributed data or median (inter-quartile range) if continuous data were skewed. Also bar graphs for categorical data such as reported contraceptive use by year of study were constructed. The main outcome of this study was current use of any contraceptive method, measured as a binary variable: “non-users” coded as “0” or “users” coded as “1”. Current use of contraception was calculated as the proportion of students who reported to have used any method of contraception in the past 12 months prior to the survey. Cross tabulations of knowledge, perceptions and attitudes towards contraceptive use and sexual behavior stratified by year of study were generated. The prevalence of contraceptive use was determined as the number of users divided by all female participants. Prevalence ratios (PRs) with their corresponding 95 % confidence intervals were used as measures of association between contraceptive use and associated factors. The PRs were obtained via a modified Poisson regression model using a generalized linear model with Poisson as family and a log link without an offset but including robust standard errors. The log-binomial model could not converge to provide an estimate of the PRs. Odds ratio could not be used as a measure of association because of the potential to overestimate the effect due to the high prevalence of the primary outcome. The year of study was considered as the main exposure variable, and adjusted for other variables including age, marital status, residence of student, religion, history of pregnancy and, beliefs and attitudes about contraceptives as potential confounders. All statistical analyses were performed in Stata version 12.1 (Statacorp, College Station. Texas).

## Results

Table 1 shows the characteristics of the study participants. A total of 1008 students were enrolled in the study. The recruitment of participants at the multiple independent academic units resulted into 35 extra participants, which was reported to the Institutional Review Board (IRB) as a protocol deviation. The mean (SD) age was 21.7 (2.3) years, with majority (87.5 %) aged 20–24 years, 38.6 % in year 2 of study, and Christian faith (Catholics 33.6 %, protestant 38.7 %) as the major reported religion. Most students were not married (87 %), just over a third (36.6 %) were resident in hostels, followed by homes (28.6 %), and only 18 % were staying at the halls of residence at the university campus.

**Table 1:** Baseline Characteristics of 1008 Female University Students, April 2014

Characteristics	Number	Percent, %
Total	1,008*	100
Age category		
18-19	65	6.4
20-24	882	87.5
25-30	61	6.1
Median Age (IQR)	21 (20, 22)	
Mean (SD)	21.7(2.3)	
Year of study		
Year 1	319	31.7
Year 2	389	38.6
Year 3+	298	29.6
Religion		
Catholics	339	33.6
Protestants	390	38.7
Muslim	90	8.9
Evangelical/SDA	189	18.8
Marital status		
Not married	878	87.5
Consensual (Co-habiting)	93	9.3
Married	33	3.3
Residence		
Home	288	28.6
Hall on Campus	188	18.7
Hostel	368	36.6
Other arrangements	162	16.1
Hall Assignment		
Africa	327	32.6
CCE	309	30.8
Mary Stuart	368	36.7
Africa	327	32.6

Note: * may vary because of missing values in some variables

### Knowledge of contraceptive and sexual reproductive health

Table 2 shows Percentage distributions of Knowledge of Contraceptive Methods and Sexual Reproductive Health. Knowledge of contraceptives was nearly universal (99.6 %). The most commonly known modern methods were pills (86.7 %) and male condoms (88.4 %), followed by injectables (50.3 %), IUDs (35 %) and implants (26.7 %), female condom (22.1 %), while withdraw (34.2 %) was the most commonly mentioned traditional methods. The commonest sources of contraceptives were Hospitals (government, 64.3 %; private, 53.6 %), clinics (general 24 %, or Contraceptives 27.4 %) and pharmacy/drugs shops (36 %). The level of knowledge was also very high regarding sexually transmitted infections (98.7 %), HIV/AIDS (99.3 %) and prevention of HIV/AIDs (98.8 %) as well as its treatment (96 %). However, the proportions of students who knew about availability of treatment for HIV and STIs within their environs was low; 44.2 % and 59.2 % respectively.

**Table 2:** Percentage distributions of Knowledge of Contraceptive Methods and Sexual Reproductive Health

			Year-1	Year-2	Year 3+
Number	1003†		319	389	298
Ever heard of contraceptive	Number	Percent "yes", (%)			
Yes	999	99.6			
Contraceptive methods known					
Modern					
Tubal ligation	99	9.8	7.5	9.3	13.1
Vasectomy	99	9.8	8.2	9.5	12.1
Pill	874	86.7	87.1	86.1	86.9
IUD	353	35.0	26.3	35.5	43.6
Injection	507	50.3	48.3	49.4	53.7
Implants	269	26.7	18.2	29.8	31.9
Male condoms	891	88.4	88.4	87.1	90.3
Female condoms	223	22.1	18.5	21.1	27.5
Diaphragm	74	7.3	5.6	5.7	11.4
Foam	35	3.5	2.5	2.1	6.4
Traditional					
Lactational Amenorrhea	36	3.6	1.9	3.1	6.0
Rhythm/moon beads	103	10.2	9.7	10.0	11.1
Withdrawal	345	34.2	31.0	36.2	35.6
Know source of contraceptives					
No	30	9 (3.0)			
Yes	961	932 (97.0)	96.2	97.9	96.6
Government					
Hospital	648	64.3	61.1	67.1	64.1
Health Centers	242	24.0	26.0	25.7	19.8
Family planning clinic	276	27.4	23.8	28.0	30.2
Outreach	22	2.2	1.6	2.3	2.7
Community distributors	41	4.1	4.4	4.4	3.4
Private providers					
Hospital/clinic	540	53.6	54.5	50.6	56.4
Pharmacy/Drug shop	363	36.0	32.0	40.1	35.2
Midwife	17	1.7	0.94	2.3	1.7
Outreaches	20	2.0	0.94	3.1	1.7
NGO community based	35	3.5	3.1	4.9	2.0
STIs Knowledge (yes)					
Heard of STIs	995	98.7	99.1	98.2	99.3
Knowledge of HIV/AIDS	1,001	99.3	99.1	99.0	100
Knowledge of HIV prevention	996	98.8	98.7	97.9	100
Heard about treatment of HIV	968	96.0	93.7	96.1	98.3
Knowledge of HIV treatment availability	445	44.2	41.4	45.0	45.6
Knowledge of STIs treatment availability	597	59.2	53.9	60.9	62.4
Heard of VCT	980	97.2	95.0	98.2	98.3

†Number is less than 1008 because of 5 missing values on some variables, Percentage are row percent of each total number of respondents per variable

### Perceptions and attitudes towards contraceptive methods by year of study

Table 3 shows perceptions and attitudes towards contraceptive methods by year of study. Overall, nearly a quarter (23.6 %) perceived that modern contraceptive services and commodities were not accessible, or that it is not easy to discuss sexual matters with partner (24.4 %). About one in five students perceived that contraceptives were not for poor people (21.3 %) or that it is wrong to use contraceptives (20.1 %). However, only 6 % believed that contraceptives were for females only. Attitudes to contraceptives being acceptable in the student community (93 %), being beneficial for males too (97.8 %), and couple counseling being able to increase male involvement in contraceptive use (96.2 %) were highly rated as "agreed". All assessed perceptions and attitudes did not significantly differ by year of study.

**Table 3:** Perceptions and Attitudes Towards Contraceptive Methods by Year of study

		Year of study			
Variable	Overall	Year −1	Year-2	Year-3+	
	N (%)*	N (%)*	N (%)*	N (%)*	P-values
Modern contraceptive services and commodities are inaccessible					
Agree	969 (23.6)	307 (25.1)	374 (22.5)	288 (23.6)	0.7289
Not easy to discuss sexual issues with partner					
Agree	684 (24.4)	184 (27.2)	274 (25.5)	226 (20.8)	0.2300
Couple counseling can improve male involvement in contraceptive use					
Agree	913 (96.2)	280 (97.5)	354 (94.6)	279 (96.8)	0.9499
Contraceptive are for females only					
Agree	971 (6.1)	308 (6.5)	376 (4.8)	287 (7.3)	0.7226
Contraceptives are acceptable in our University community					
Agree	975 (93.0)	309 (94.8)	377 (94.4)	289 (89.3)	0.6185
Contraceptives benefits males too					
Agree	989 (97.8)	311 (96.7)	386 (98.4)	292 (97.9)	0.8928
Contraceptives is not for the poor					
Agree	986 (21.3)	311 (22.8)	384 (21.1)	291 (19.9)	0.4829
It is wrong to use contraceptives					
Agree	989 (20.4)	314 (20.1)	383 (20.6)	292 (20.5)	0.9019

*N = Total number of respondents with information on each variable, while % = percentage of respondents with “Agree” for each variable

### Contraceptive use, sexual behavior and pregnancy

Table 4 shows contraceptive use, sexual behavior and pregnancy rates. Overall half (51.2 %) of the students were currently in sexual relationships, with slight increments in the proportions across the year of study. Nearly 70 % had ever engaged in sexual intercourse while 62.1 % reported sexual intercourse in the past 12 months. Over half (55.1 %) had ever used any method to prevent pregnancy while only 46.6 % were currently using contraceptives with male condoms being the most commonly mentioned methods used (34.5 %). Ever being pregnant or knowledge of a pregnant friend significantly varied by year of study; overall, 9 % reported ever being pregnant, 4.1 % (year-1), 9.8 % (year2) and 13.1% (year3+); (p = 0.003) while a third (33.8 %) knew of a pregnant friend 16.3 % (year-1), 38.9 % (year2) and 46.0 % (year3+), p < 0.001. Only 2 % of students reported being pregnant; higher among year 3+ compared to those in year-1. About 40 % of ever pregnant respondents reported ever trying to terminate a pregnancy.

**Table 4:** Contraceptive Use and Sexual Behaviors by Year of Study

Variable	Overall	Year-1	Year-2	Year-3+	P-values
	N (%)**	N (%)**	N (%)**	N (%)**	
Current Contraceptives use					
Yes	986 (46.6)	317 (41.6)	381 (50.9)	288 (46.2)	0.4643
Currently in sexual relationship					
Yes	1006 (51.2)	*319 (45.5)*	389 (54.5)	298 (53.0)	0.2730
Ever had sexual intercourse					
Yes	833 (69.7)	*249 (62.2)*	326 (72.7)	258 (73.3)	0.2588
Had sex in past 12 months					
Yes	850 (62.1)	*261 (55.9)*	326 (65.6)	263 (63.9)	0.3664
Ever used any method to prevent pregnancy					
Yes	985 (55.1)	312 (47.4)	376 (58.8)	297 (58.6)	0.1313
Current Contraceptives method					
None	*527 (53.4)*	*185 (58.4)*	*187 (49.1)*	*155 (53.8)*	0.5809
Traditional	*32 (3.2)*	*14 (4.4)*	*10 (2.6)*	*8 (2.8)*	0.7053
Condoms-only	*340 (34.5)*	*98 (30.9)*	*149 (39.1)*	*93 (32.3)*	0.8487
Modern with/out condoms	*87 (8.8)*	*20 (6.3)*	*35 (9.2)*	*32 (11.1)*	0.4009
Know a pregnant friend					
Yes	*1005 (33.8)*	*319 (16.3)*	*388 (38.9)*	*298 (46.0)*	*<0.001*
Ever been pregnant					
Yes	*1004 (9.0)*	*318 (4.1)*	*388 (9.8)*	*298 (13.1)*	*0.0003*
Ever tried to terminate pregnancy					
Yes	*90 (40.0)*	*13 (38.5)*	*38 (36.8)*	*39 (43.6)*	0.7280
*Currently pregnant (self-reported)					
Yes	1005 (2.0)	*319 (0.63)*	*388 (2.1)*	*298 (3.4)*	*0.0174*

** *N = Total number of respondents with information on each variable, while % = percentage of respondents with “Agree” for each variable

### Current contraceptive use by sexual active students

**Fig. 1:**
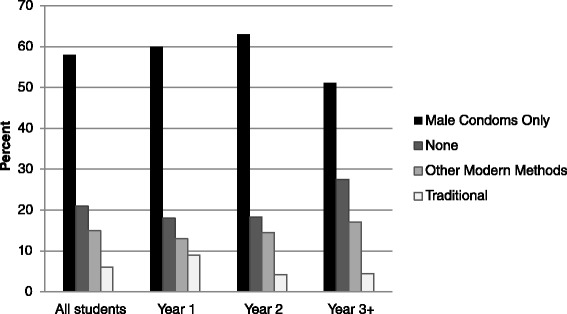
Current Use of Contraceptive Methods by 626 Sexually Active Students

Figure 1 shows distribution of current contraceptive use by year of study. Among the sexually active students, male condoms 58 %, (n = 363) or other modern contraceptives 15 % (n = 93) where the most commonly mentioned methods, while 6 % (n = 37) only reported use of a traditional method. This pattern was similar across year of study. Although respondents were sexually active, about 1 in 5 (22 %) did not use any contraceptive, and this was most common in year-3+ (27.5 %) compared to either year 1 (18.4 %) or year 2 (18.3 %).

### Factors associated with contraceptive use

Table 5 shows the modified Poisson multivariable regression analysis of factors associated with contraceptive use. Compared to year-1, year-2 had a 22 % higher prevalence of contraceptive use. Contraceptive use was 3 % higher if a student was one year older than their counter-part adjusting for other factors.

**Table 5:** Factors Associated with Modern Contraceptives Use Among Female University Students

Variable	Crude PR (95 % CI)	Adjusted PR (95 % CI)	P-value
**Year of Study**			
Year-1	1.0	1.0	
Year-2	**1.28 (1.07,1.53)**	**1.22 (1.02,1.45)**	**0.029**
Year 3+	1.17 (0.96,1.42)	1.09 (0.89, 1.34)	0.388
**Age (years)**	**1.05 (1.04,1.08)**	**1.03 (1.00, 1.06)**	**0.027**
**Marital status**			
Not married	1.0	1.0	
Consensual union	**1.91 (1.65,2.21)**	**1.75 (1.50, 2.05)**	**<0.001**
Married	1.84 (1.43,2.36)	1.32 (0.92, 1.90)	0.132
**Residence**			
Home	1.0	1.0	
University hall	0.80 (0.62,1.02)	0.84 (0.65, 1.07)	0.161
Hostel	1.07 (0.90,1.28)	1.08 (0.91, 1.28)	0.392
Other arrangements	1.23 (0.99,1.51)	1.06 (0.87, 1.29)	0.566
**Religion**			
Catholics	1.0	1.0	
Protestants	1.13 (0.96,1.33)	1.16 (0.99, 1.36)	0.063
Muslim	1.19 (0.93,1.51)	1.17 (0.93, 1.48)	0.189
Evangelical/SDA	**0.62 (039,0.50)**	**0.65 (0.50, 0.84)**	**0.001**
**Ever been pregnant**			
No	1.0	1.0	
Yes	**1.66 (1.39,1.98)**	1.18 (0.93, 1.48)	0.166
**Contraceptive methods are for females only**			
Disagree	1.0	1.0	
Agree	**1.33 (1.04,1.69)**	**1.41 (1.10, 1.18)**	**0.007**
**It is wrong to use contraceptives**			
Disagree	1.0	1.0	
Agree	**0.67 (0.54,0.84)**	**0.67 (0.55, 0.83)**	**<0.001**

Bold type numbers indicate statistical significance at <0.05 level

*PR*:Prevalence ratio

The prevalence of contraceptive use was 75 % higher among married compared to none married, and 35 % lower in the Evangelical or SDAs compared to the Roman Catholic students. Perception of contraceptive methods being for females only was associated with a 41 % higher prevalence of contraceptive use relative too those who disagreed, while students who perceived it as being wrong to use contraceptives has a 33 % lower prevalence of use relative to those who had a favourable perception.

## Discussion

The study assessed the knowledge, attitudes, perceptions and use of contraceptives among female undergraduate students of Makerere University. Our findings show that knowledge was universal, but contraceptive use was suboptimal. The most commonly known and used methods were the male condoms and oral pills, but knowledge of the female condom was very low. Positive perceptions and attitudes were strongest on couple counseling acceptability of contraceptives at the university and benefits of contraceptives to males. Negative perceptions about contraceptives being for the poor or their use being wrong were mentioned. High level of contraceptive knowledge does not translate into actual use in this study or from other studies [14, 15]. Religious beliefs as evidenced by lower use of contraceptives by evangelicals or Seventh Day Adventist have a clear negative influence of utilization; this has also been shown in other university setting in western Uganda [16]. Religious and moral beliefs clearly overlap and need further exploration in a University setting.

The level of knowledge about contraceptives was found to be lower in similar African university settings, ranging 53.3 % to 86.3 % [17–21]. It is possible that even though students were universally aware of a range of contraceptive methods and knew where to get the services, they may have faced other obstacles that we did not directly measure in our study. For example, previous studies done in Uganda have highlighted social-cultural factors as critical barriers to contraceptive use in young females [15, 22]. Nalwadda and colleagues (2010) conducted a study in rural Uganda that specifically showed that societal norms such as condemning early engagement in sex, pregnancy and use of contraceptives among young unmarried girls presents a major obstacle to contraceptive use [23]. In our urban-based study, similar socio-cultural factors still have a lot influences leading to poor uptake of contraceptive among female students. In designing youth-friendly interventions it is imperative that efforts should be geared towards disseminating specific contraceptive information and education that is culturally-sensitive to the local setting.

Students knew the main sources of contraceptives services as government hospitals and private clinics but we did not verify if these were there major sources of contraceptive information. Awareness of sources was shown to reduce barriers and improve contraceptive use among female students in the University of Lesotho [1]. However, a study done in Ethiopia that evaluated major sources of information about contraceptives found out that a high knowledge of sources was not enough to result into actual use, rather the students needed more information about freely available contraceptive services [19, 24]. These observations suggest that reproductive health education programs should offer accurate and comprehensive information while building skills for negotiating safer sexual behaviours [7].

Overall the students had positive attitudes and perceptions towards using contraceptives. However, negative perceptions and attitudes existed about accessibility to contraceptive services; these included discussion of sexual issues with partner, contraceptives being for the poor and wrong perceptions about contraceptive use. A study in Nigeria reported that a high proportion of students perceived contraceptive use as bad because they believed it caused infertility [25]. However, studies have reported positive attitudes specifically toward emergency contraceptives among students at Jimma and Adama university in Ethiopia [26, 27] and negative attitudes toward emergency contraceptives increasing promiscuity in female students of Trinidad and Tobago [28].

Sexual activity among adolescents and young females is often associated with a greater risk for unintended pregnancies [2]. In our study, nearly 70 % of the students were sexually active in the past 12 months. This finding supports results from the Uganda demographic health survey showing that premarital sex is common, with at least one in five young females aged 15–24 being sexually active [9]. Young females joining universities often become sexually active partly due to peer-pressures, alcohol use, or as result of a perceived sense of being in control of their social lives [26]. Similar levels of sexual activity were reported from other studies done among female university students of the same age group [29]. In contrast, some earlier studies done in Africa showed lower levels of sexual activity among university students, these ranged from 14 % to 48 % [20, 25, 26, 30, 31]. The differences in levels of sexual activity may be due to temporal events like increased sexual reproductive health over time or could be explained by differences in religious and cultural beliefs surrounding premarital sex [32].

Contraceptive use of any method among the university students was 46.6 % which is nearly twice as high as the contraceptive prevalence of Uganda. On the other hand, the rate of use is lower than 79 % contraceptive used among females that was reported from a study done in Mbarara University in Uganda [16] and in other university students in Lesotho, Kenya and Ethiopia [1, 14, 33]. Factors that were significantly associated with contraceptive use in this study are consistent with findings from a recent survey in a nationally representative in Ugandan women [22]. The high level of sexual activity and the risk of unintended pregnancies point to a need to promote sexual and reprodcutive health sevices in this setting. Male condoms were the most common contraceptive methods followed by oral pills among the sexually active students. Similar findings have been obseved elsewhere in both the developed [13, 31, 34] or developing countries [1, 25, 27, 28, 35]. However methods such as oral pills and injectable contraceptives have also been mentioned as being common in some settings such as Adama University, Ethiopia [27].

Among the sexually active students, one in five were engaging in unsafe sex suggesting high risk to unintended pregnancies and sexually transmitted diseases including HIV infection. Unsafe sexual practices are common among young adults especially in universities and other higher educational institutions [10, 11, 13]. The reported prevalence of unsafe sex may be higher in this setting if some students also use emergency contraceptives for prevention of pregnancy. Previous studies elsewhere have shown high level of unsafe sex [31, 33] in similar settings. However, our study did not specifically evaluate the use of emergency contraceptives.

The prevalence of reported pregnancy at the time of survey was low (2 %) but probably under-reported due to associated stigma among unmarried pregnant women. However, 9 % of the sexual active students reported being ever pregnant. Among the ever-pregnant females, 40 % had ever tried to terminate the pregnancy suggesting a high level of both unmet need for contraceptive and unintended pregnancy in this setting. The reported 40 % ever pregnant who tried to terminate a pregnancy may be an under estimate because abortion is illegal in Uganda, and can be very stigmatizing. Similar findings about abortion have been observed in Mexico public university among first year medical students where half (52 %) of the ever been pregnant female students performed unsafe abortions [36], and in Addis Ababa University Ethiopia 9 in 10 (90 %) of the ever pregnant students terminated their pregnancies with induced abortion [20].

### Study strengths and limitations

Although the findings from this study are consistent with results from other university settings, we encountered some limitations. The planned random sampling based on the sampling frame from the University’s academic registrar was dropped in favor of convenience sampling. However, interviewers were assigned/stationed at multiple academic units and asked to as much as possible randomly select participants without any specific characteristics. Some of the information especially on issues such as abortion may be under-reported because abortion is illegal and stigmatizing. Also pregnancy among unmarried females is culturally unacceptable thus leading to a potential for under-reporting. However, the study team recruited and trained experienced interviewers within the age range of the respondents as a way of minimizing reporting bias. The consistence in the contraceptive use indicators with other studies and the nationally representative samples such as the Uganda demographical and health surveys provide credence and strength to findings observed in this study.

## Conclusions

Knowledge, perceived acceptability and benefits of contraceptive use were nearly universal, but contraceptive use was suboptimal in this setting. Ever trying to terminate a pregnancy was common and a clear indicator of unintended pregnancies. Religious beliefs and misconceptions of need for contraceptive are key determinants of poor uptake of contraceptives.

### Recommendations

Interventions that promote translation of knowledge into proper sexual and reproductive health practices are urgently needed. The high rate of unintended pregnancy as measured by ever-attempt to terminate a pregnancy needs to be addressed through increased counseling and information about contraceptives, and accessible and of contraceptive services to students who desire to use them.

Religious leaders may have to be engaged to discuss issues of sexual and reproductive health and how to avert the unintended pregnancies. The leadership of evangelical and SDA faith can be an important stakeholder in this endeavor.
